# To the Question on the Use of Multivariate Analysis and 2D Visualisation of Synchrotron ATR-FTIR Chemical Imaging Spectral Data in the Diagnostics of Biomimetic Sound Dentin/Dental Composite Interface

**DOI:** 10.3390/diagnostics11071294

**Published:** 2021-07-19

**Authors:** Pavel Seredin, Dmitry Goloshchapov, Vladimir Kashkarov, Yuri Ippolitov, Ivan Ippolitov, Jitraporn Vongsvivut

**Affiliations:** 1 Solid State Physics and Nanostructures Department, Voronezh State University, University sq.1, 394018 Voronezh, Russia; goloshchapovdl@gmail.com (D.G.); kash@phys.vsu.ru (V.K.); 2 Department of Pediatric Dentistry with Orthodontia, Voronezh State Medical University, Studentcheskaya st. 11, 394006 Voronezh, Russia; dsvgma@mail.ru (Y.I.); stomat@vmail.ru (I.I.); 3 Infrared Microspectroscopy (IRM) Beamline, ANSTO—Australian Synchrotron, 800 Blackburn Road, Clayton, VIC 3168, Australia; jitrapov@ansto.gov.au

**Keywords:** synchrotron ATR-FTIR chemical imaging visualisation, multivariate analysis, diagnostics of biomimetic interface, hybrid layer formation

## Abstract

In this short communication, we provide information on the use of the hierarchical cluster analysis of synchrotron ATR-FTIR 2D chemical imaging spectral data as a useful and powerful approach to the microspectroscopic diagnostics of molecular composition in the hybrid sound dentin/dental composite interfaces and materials, including ones developed with the use of biomimetic strategies. The described diagnostic approach can be successfully transferred to the analysis and visualisation of 2D spectral data, collected using laboratory Raman and FTIR microspectroscopy techniques.

## 1. Introduction

The personalised approach in dentistry uses new molecular tools, nanotechnology, and visualisation setups to allow the dentists and scientists to effectively control the function and aesthetics of restorations. This approach uses durable and modern smart materials to maintain the health and vitality of the patient’s natural dental tissue [1].

It’s well known that forming a stable bound between the restorative composite material and the human hard dental tissue is due to forming a hybrid layer [2,3]. The existing strategies of hybrid layer formation [4,5] are gradually improved. As concerning biomimetic similarity [4], these concepts are based on the new approaches and achievements in nanotechnologies [6,7]. Materials and bonding systems applied in dentistry nowadays have different compositions and are fabricated by different technologies, while some of them involve nanomaterials that are used to get the best reconstruction of the formation of the biogenic tissues [8]. There is an assumption that the qualitative bio-interface between synthetic material and natural hard tissue can be realised based on biomimetic strategy supposing the formation of the intermediate layer with the use of nanocrystalline carbonate-substituted hydroxyapatite (nano-c-HAp) and several polar amino acids [6,9,10]. Determination of the hybrid layer’s integration mechanisms (molecular bounding), evaluation of its chemical composition and quality can be successfully performed based on FTIR chemical mapping technique [11,12]. FTIR maps of functional groups (FTIR chemometrics) can reveal the chemical composition throughout a material/tissue and the spatial variation of the chemical properties within a small volume of biomaterial [11,13,14]. Spectral bands used in chemometrics imaging often overlap with other vibrations. Therefore, the use of only FTIR chemometrics imaging mode does not always enable observation of the fine spectral differences that can be a key point to the evaluation of molecular interaction. Therefore, the use of the multivariate statistical analysis results for the array of the collected spectral data with the 2D visualisation of results should become an effective biochemical fingerprints instrument for the diagnostics and evaluation of formation of the biomimetic dentin/dental composite interface [15]. 

## 2. Materials and Methods

For sample creation, five healthy molars extracted from patients aged 18–25 due to orthodontic indications at the Burdenko Voronezh State Medical University dental clinic were used in this study. The patients were physically healthy based on outpatient records and had no unhealthy behaviours, including smoking. Teeth were collected following the relevant regulations. Informed consent was obtained from all participants, and data collection and handling followed the Helsinki declaration. 

Then with an Er:YAG pulse laser (wavelength 2940 nm, pulse duration 75–500 μs, frequency of 10–50 Hz, Max power = 8 W) in the dental tissue, a cavity with a cylindrical shape was formed. In order to form a hybrid biomimetic interface on the surface of the cavity, a layer of bioprimer, adhesive, and compomer material DyractXP was deposited. Powder-like nanocrystalline carbonate-substituted hydroxyapatite was preliminarily introduced into adhesive (1 mL adhesive–0,01 g of nano-c-HAp). Nano-c-HAp was obtained from bird eggshells via liquid-phase synthesis as it was previously described [16]. Filling in of adhesive with nano-c-HAp should facilitate the elimination of stresses formed during polymerisation, as it leads to the increase of the layers’ hardness [17]. Mixing of the components of nano-c-HAp and adhesive was performed using ultra-sound the homogenizer QSonica 55 W. After the photopolymerisation process for the interface materials, we prepared planar segments of the samples. Flat-parallel segments of the restored tooth samples were prepared for FTIR micro-mapping as previously described [18,19]. Tooth samples were separated using a low-speed, water-cooled diamond saw, and the resulting hard tissue layers were gently sanded and polished using a diamond abrasive.

The samples were studied using the synchrotron FTIR technique at the Infrared Microspectroscopy (IRM) beamline (Australian synchrotron), using a Bruker Vertex 80 v spectrometer coupled with a Hyperion 3000 FTIR microscope. A subsequent synchrotron macro ATR-FTIR mapping measurement was performed on specific areas of interest (15 μ × 45 μ) found in the prior overview map. All the synchrotron FTIR spectra were recorded within a spectral range of 3800–700 cm^−1^ using 4-cm^−1^ spectral resolution. The unique combination of the high refractive index property of the Ge ATR crystal and the high NA objective used in this device, when coupled to the synchrotron-IR beam, allows surface characterisation of the teeth slices to be performed at a high lateral resolution (500 nm) and without scattering artifacts. 

The spectral data (FTIR-maps) collected array was estimated using the hierarchical cluster analysis (HCA) technique. HCA is a multivariate statistical approach for the classification of spectroscopic analysis. Using the HCA technique, one can identify the areas in the structure of a sample by their spectral response. The areas where the points show similar spectral responses demonstrate minimal intra-cluster spectral differences. At the same time, maximal inter-cluster differences should be observed for the areas with different spectral responses [12]. While performing HCA for processing the raw spectral data, we applied second derivative and vector normalisation in the range of FTIR-spectrum at 1800 cm^−1^–950 cm^−1^. The basic vibrations characteristic for the investigated materials were arranged in this range. Smoothing of the spectra was performed by 17 points. 

## 3. Results

**Figure 1:**
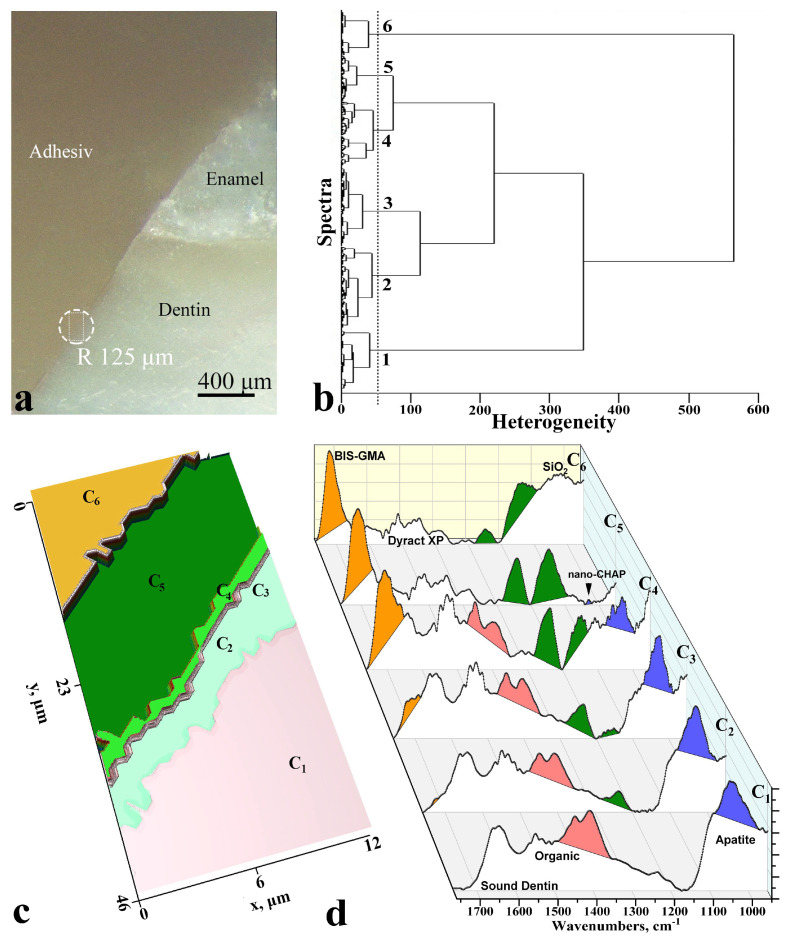
The use of multivariate analysis of synchrotron ATR-FTIR chemical imaging spectral data and its 2D visualisation for the diagnostics of biomimetic sound dentin/dental composite interface for the typical sample. (**a**)-optical images of interface area, (**b**)-dendrogram of heterogeneity, (**c**) colour coding of the cluster analysis results, (**d**)-the averaged FTIR-spectra of each cluster.

The representative optical image of the interface area is presented in Figure 1a. Results of cluster analysis (dendrogram and clustered 2D FTIR image of the interface) are presented in Figure 1b,c.

Results of the cluster analysis (Figure 1c) are represented with the use of colour coding. All points in the scanned area have their own colour within the boundaries of one cluster.

One can easily see that there are six clusters in the area of interface sound dentin/adhesive/dental composite (Figure 1c). The width of the formed inter-boundary area was ~20 μm. The lateral arrangement of the clusters enables the probing of characteristic regions in the interface. In order to determine the detailed difference at the boundary of the interface and the following chemical differentiation of these areas, we extracted the averaged spectra of each cluster (presented in Figure 1d). In 1,d figure demonstrating FTIR spectra of the clusters were highlighted in colour in the molecular groups where the greatest changes were observed. These can be related to the region 1500–1700 originated from amide I and II peaks from dental proteins. It also included the spectral band at 1110–960 cm^−1^ associated with PO_4_ vibrations from the mineral component of dentine apatite and the SiO_2_ group in the Dyract XP compomer and spectral band at 1750–1700 cm^−1^ assigned to the vibrations of the ester group (-COOCH3) in the Bis-GMA adhesive and Dyract XP compomer. Analysis of the spectral data was performed based on a series of references where materials applied for obtaining biomimetic interface were studied by FTIR technique [11,20,21,22,23]. 

## 4. Discussion

Chemical imaging performed based on hierarchical cluster analysis enabled visualising and identifying the regions in the hybrid layer of biomimetic sound dentin/dental composite interface. Our investigations show that the ultimate clusters of the biomimetic sound dentin/dental composite interface represent the 3D area of sound dentin (cluster C_1_) and material DyractXP (cluster C_6_) [24]. Cluster C_2_ is the area of partially disorganised dentin. The appearance of the narrow region (~1–2 μm) in the transition area of the biointerface corresponding to cluster C_3_ is a result of the treatment of dentin surface modified with a dentin-conditioner and bioprimer. Characteristic vibrations are detected in the spectrum of the C_4_ cluster (Figure 1d) that are associated with bioprimer and modified adhesive. Cluster C_5_ is an area where distinctions of the modified adhesive where are observed. In the range of 1025 cm^−1^ of FTIR-spectrum (Figure 1d) in C_5_ cluster, low-intensive distinctions are observed associated with the most intensive mode of carbonate-substituted nano-sized hydroxyapatite applied for the modification of adhesive. 

Microspectroscopically visualised 2D zones in the biomimetic hybrid layer are distinguished by the characteristic spectra. Using these spectra, it is possible to monitor the sound dentin/dental composite interface state in the future and develop novel diagnostic instruments and technological improvements for restorative dentistry. 

As it follows from the visualisation of FTIR chemometric data and cluster analysis (Figure 1c,d), chemically uniform composition over the depth and width is characteristic for the fabricated biomimetic interface as in the dentin area as in the hybrid layer. Involvement of nanocrystalline carbonate-substituted hydroxyapatite nano-c-HAP with a structural and morphological organisation similar to the natural apatite of the dental tissue into the bonding system enabled formation of a biomimetic hybrid interface based on polymer, amino acids and a resin substituting natural dentin tissue at the boundary of sound dentin/dental composite. Such an approach already provided some positive results allowing formation of the stable bounds between adhesive and dentin.

Innovations related to the introduction of new visualisation approaches in dentistry may soon change the paradigm in the dental field, thereby achieving a high level of personalisation. As a result, the development of new and smart solutions in nano dentistry [2] will enable the effective prevention of secondary caries around restorations.

## 5. Conclusions

Thus, summarising the obtained results, it is possible to state with confidence that the use of the hierarchical cluster analysis of synchrotron ATR-FTIR chemical imaging spectral data is a favorable and powerful approach employed in microspectroscopic diagnostics. This is done with a high spatial resolution of molecular composition in hybrid sound dentin/dental composite interfaces and materials, including ones designed with the use of biomimetic strategies. The described diagnostic approach can be successfully transferred to the analysis and visualisation of 2D spectral data collected using laboratory Raman and FTIR microspectroscopy.

## Data Availability

The data that support the findings of this study are available from the corresponding author upon reasonable request.
